# Characterizing the Typical Information Curves of Diverse Languages

**DOI:** 10.3390/e23101300

**Published:** 2021-10-02

**Authors:** Josef Klafka, Daniel Yurovsky

**Affiliations:** Department of Psychology, Carnegie Mellon University, 5000 Forbes Ave, Pittsburgh, PA 15213, USA; jlklafka@gmail.com

**Keywords:** communication, typology, language development

## Abstract

Optimal coding theories of language predict that speakers will keep the amount of information in their utterances relatively uniform under the constraints imposed by their language, but how much do these constraints influence information structure, and how does this influence vary across languages? We present a novel method for characterizing the information structure of sentences across a diverse set of languages. While the structure of English is broadly consistent with the shape predicted by optimal coding, many languages are not consistent with this prediction. We proceed to show that the characteristic information curves of languages are partly related to a variety of typological features from phonology to word order. These results present an important step in the direction of exploring upper bounds for the extent to which linguistic codes can be optimal for communication.

## 1. Introduction

One of the defining features of human language is its power to transmit information. We use language for a variety of purposes like greeting friends, making records, and signaling group identity. These purposes all share a common goal: Transmitting information that changes the mental state of our listener [1]. For this reason, we can describe language as a cryptographic code, one that allows speakers to turn their intended meaning into a message that can be transmitted to a listener, and subsequently converted by the listener back into an approximation of the intended meaning [2].

How should we expect this code to be structured? If language has evolved as a code for information transmission, its structure should reflect this optimization [3], and the optimal code would have to work with two competing pressures: (1) the need for listeners to decode the speaker’s message easily and successfully and (2) for the speaker to code a message to transmit it to a listener with minimal effort and error. A fundamental constraint on both of these processes is the linear order of spoken language–sounds are produced one at a time, so each is perceptually unavailable once it is no longer being produced.

Humans accommodate this linear order constraint through incremental processing: People process speech continuously as it arrives, predicting upcoming words and building expectations about the meaning of an utterance in real time rather than at its conclusion [4,5,6]. This solution creates a new guidance for speakers. Since prediction errors can lead to severe processing costs and the listeners’ difficulty integrating new information, speakers should seek to minimize prediction errors. However, the cost of producing more predictable utterances entails using more words. Thus, the most efficient strategy for speakers to minimize production costs is to produce utterances that are just at the prediction capacity of listeners but do not exceed it [7,8,9]. In other words, a speaker should maintain a constant transmission of information that is as close to the listener’s fastest decoding rate as possible. The hypothesis that speakers follow this optimal strategy is known as the “Uniform Information Density (UID)” hypothesis.

Using information theory, a mathematical framework for formalizing predictability, researchers have tested and confirmed this optimal coding prediction across several levels and contexts in language production. For example, Genzel and Charniak [8] provided a clever indirect test of UID across sentences in a paragraph. They showed that the predictability of successive sentences, when analyzed in isolation decreases, which would be expected if readers used prior sentences to predict the content of subsequent sentences. Thus, based on the increasing amount of context, they found that total predictability remained constant. At the level of individual words, Mahowald et al. [10] showed that speakers used shorter alternatives of more predictable words, maximizing the amount of information in each word while minimizing the time spent on those words.

Other research has suggested that efficient encoding influences how speakers structure units between words and sentences. The inclusion of complementizers in relative clauses [11] and the use of contractions [12] are two situations in sentence formation where speakers can omit or reduce words to communicate more efficiently and maximize use of the communication channel without exceeding the listener’s capacity.

How languages evolve is shaped by efficient communication as well. Piantadosi et al. [13] showed that the more easily predictable words in a language tend to become shorter over time, maximizing the amount of information transmitted per second. Semantic categories of words across languages can also evolve to be structured efficiently. Categories such as kinship terms [14] maintain a trade-off between informativeness and complexity. Structure in language evolved from a trade off between efficient and learnable encoding on the one hand and an expressive and descriptive lexicon on the other [15]. Languages may come to describe efficiently the particular environment in which they are spoken over the course of evolution: features of the world that are relevant to speakers become part of a language, while irrelevant features are disregarded [16].

However, speakers are still bound by syntactic rules. While complementizers are often optional, determiners are not. Similarly, speakers may have a choice about which of several near-synonyms they produce, but they cannot choose the canonical order of subjects, verbs, and objects. Properties of a language, like canonical word order, impose top-down constraints on how speakers can structure what they say. While speakers may produce utterances as uniform in information density as their languages will allow, these top-down constraints may create significant and unique variation across languages.

How significant are a language’s top-down constraints on determining how its speakers structure their speech? Yu et al. [17] analyzed how the information in words of English sentences of a fixed length varies with their order in the sentence (e.g., first word or second word). They found a surprising non-linear shape, and argued that this shape may arise from top-down grammatical constraints. In this paper, we replicate their analysis and extend their ideas. We ask: (1) Whether this shape is affected by the amount of context in prediction, (2) whether this shape varies across written and spoken language, and (3) whether this shape is broadly characteristic of a diverse set of languages or varies predictably from language to language. We found that languages are characterized by highly reliable but cross-linguistically variable information structures that co-vary with top-down linguistic features. However, using sufficient predictive context partially flattens these shapes across languages in accord with predictions of the UID hypothesis.

## 2. Study 1: The Shape of Information in Written English

Genzel and Charniak [8] performed an influential early test of the Uniform Information Density hypothesis, by analyzing the amount of information in successive sentences in a corpus. They reasoned that if speakers kept the amount of information in each sentence constant, but readers of the text processed each successive sentence in the context of the prior sentences, then a predictive model that does not have access to this prior context should find each successive sentence more surprising. To test this hypothesis, they used a simple n-gram model in which the surprisal of each word is computed as the probability of it following each of the prior n-words. They found that the surprisal-per-word of sentences later in an article was higher than for earlier sentences.

Yu et al. [17] applied this same logic to each word in a sentence. They reasoned that if speakers were processing each word in the context of all prior words in the sentence, but their predictive model ignored this context by considering the base-rate entropy of each word, they would observe the same monotonically increasing surprisal as a function of word position within each sentence. However, this is not what they observed. Instead, they found a characteristic three-step distribution: Words early in an utterance had very low information, then information was constant throughout most of the utterance; then there was a slight dip; finally there was a steep rise for the final word. Yu et al. [17] interpreted this uneven information curve as evidence against the UID Hypothesis. Unlike Genzel and Charniak’s results, information plateaued in the middle of sentences and then dipped instead of rising throughout.

However, Yu et al. [17]’s analysis left some open questions. First, they used an unusual metric to quantify the information in each word. They computed the average surprisal of all the words in a given word position weighed by their frequency of appearance in that position. Their formula is given by $H\left(X\right)=-\sum_{w}P\left(w\in X\right)\log P\left(w\right)$, where *X* is a word position (e.g. first, fifth, final) and *w* is a word occurring in position *X*. If the goal is to consider the surprisal of each word in a model that does not use prior context, then the model should not consider sentence position either. Second, with the exception of the small dip that appears near the end of the sentence, the shape is roughly consistent with the predicted monotonically increasing per-word surprisal. Third, it is difficult to know whether the characteristic shape generalizes across sentences of all lengths rather than just the three particular lengths that Yu et al. [17] analyzed. Finally, it would be ideal to estimate the characteristic information profiles of sentences when context is considered, not just in the absence of the context of prior words; that is, ideally we could observe the uniform surprisal of words directly for a model of a reader and not just indirectly for a model of a context-less reader.

In Study 1, we attempted to resolve these issues. We first replicated Yu et al.’s analysis using a standard unigram language model and then used a trigram model to show that information in English sentences is significantly more uniform when words are processed in context. Finally, we introduced a method for aggregating across sentences of different lengths to produce a single characteristic profile for English sentences and show that they are broadly consistent with a Uniform Information Density prediction akin to Genzel and Charniak [8].

### 2.1. Method

#### 2.1.1. Data

Following Yu et al. [17], we selected the British National Corpus (BNC) to estimate the information in English sentences [18]. The BNC is an approximately 100 million word corpus consisting mainly of written (90%) with some spoken transcriptions (10%) of English collected by researchers at Oxford University in the 1980s and 1990s. The BNC is intended to be representative of British English at the end of the 20th century and contains a wide variety of genres (e.g., newspaper articles, pamphlets, fiction novels and academic papers).

**Figure 1:**
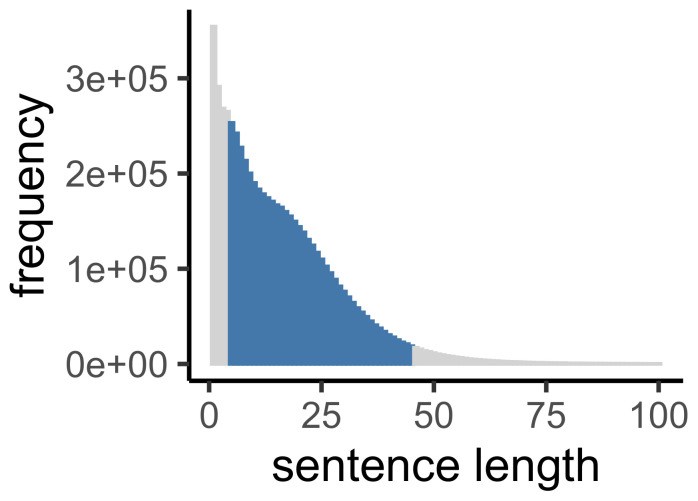
The distribution of sentence lengths of in the British National Corpus (BNC). We analyzed sentences of 5–45 words (colored).

We began with the XML version of the corpus, and used the justTheWords.xsl script provided along with the corpus to produce a text file with one sentence of the corpus on each line. Compound words (like “can’t”) were combined, and all words were converted to lowercase before analysis. This produced a corpus of just over six million utterance of varying lengths. From these, we excluded utterances that were too short to allow for reasonable estimation of information shape (fewer than 5 words), and utterances that were unusually long (more than 45 words). This exclusion left us with 89.83% of the utterances (Figure 1).

#### 2.1.2. Models

To estimate how information is distributed across utterances, we computed the lexical surprisal of words in each sentence position under two different models. First, we estimated a unigram model which considered each word independently. This unigram surprisal measure was a direct transformation of the word’s frequency and thus less frequent words were more surprising.

surprisal(word)=−logP(word)

Second, we estimated a trigram model in which the surprisal of a given word ($w_{i}$) encoded how unexpected it was to read after reading the prior two words ($w_{i-1}$ and $w_{i-2}$):

$$\mathrm{surprisal}\left(w_{i}\right)=-logP\left(w_{i}|w_{i-1},w_{i-2}\right)$$

This metric encodes the idea that low-frequency words in isolation (e.g., “meatballs”) may become much less surprising in certain contexts (e.g., “spaghetti and meatballs”) but more surprising in others (e.g., “coffee and meatballs”). Because the problem of estimating probabilities grows combinatorially with the number of prior words due to sparsity, we chose a trigram rather than a quadgram model as in Genzel and Charniak [8]. In practice, trigram models perform well as an approximation (see e.g., [19,20]).

We estimated these models using the KenLM toolkit [21]. Each utterance was padded with a special start-of-sentence token “$\langle s\rangle$” and end of sentence token “$\langle /s\rangle$”. Trigram estimates did not cross sentence boundaries, for example: the surprisal of the second word in an utterance was estimated as $\mathrm{surprisal}\left(w_{2}\right)=-P\left(w_{2}|w_{i},\langle s\rangle \right)$.

Naïve trigram models will underestimate the surprisal of words in low-frequency trigrams (e.g. if the word “meatballs” appears only once in the corpus following exactly the words “spaghetti and”, it is perfectly predictable from its prior two words). To avoid this underestimation, we used modified Kneser–Ney smoothing as implemented in the KenLM toolkit [21]. Briefly, this smoothing technique discounted all n-gram frequency counts, which reduced the impact of rare n-grams on probability calculations, and interpolated lower-order n-grams, which were weighted in the calculations according to the number of distinct contexts in which they occurred as a continuation. For example, “Francisco” may be a common word in a corpus, but likely only occurred after “San” as in “San Francisco”, so it received a lower weighting. For a thorough explanation of modified Kneser–Ney smoothing, see Chen and Goodman [19].

To prevent overfitting, we computed the surprisal of words in sentences using cross-validation. We divided the corpus into 10 sub-corpora of equal length. For each sub-corpus, we fit the unigram and trigram models on all other subcorpora, and then used this model to estimate the surprisal of words in this corpus.

#### 2.1.3. Characteristic Information Curves

**Figure 2:**
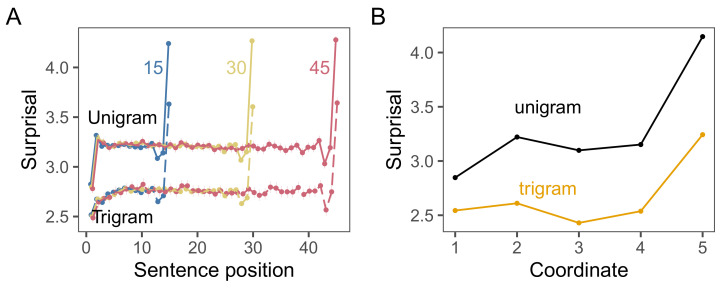
(**A**) Surprisal by sentence position of length 15, 30, and 45 sentences in the British National Corpus under unigram and trigram surprisal models. Error bars indicate 95% confidence intervals (tiny due to sample size). (**B**) Characteristic information curves produced by the DBA algorithm averaging over all sentence lengths in each corpus.

To develop a characteristic information curve for sentences in the corpus, we needed to aggregate sentences that varied dramatically in length (Figure 2A). We used Dynamic Time Warping Barycenter Averaging (DBA), an algorithm for finding the average of sequences that share an underlying pattern but vary in length [22].

Dynamic time warping is a method for calculating an alignment between two sequences in which one can be produced by warping the other. Canonically, there is a template sequence (e.g., a known vowel’s acoustic profile, a known walker’s motion-capture limb positions) and a set of unknown sequences that may be new instances of the template sequence produced by perturbations in speed or acceleration (e.g., extending pr shortening the vowel, walking faster or slower). Dynamic time warping works by finding a partial match between the known template and unknown instance under the constraint that each point in the instance must come from a point in the template, and that the ordering of the points must be preserved; however, multiple points in the sequence can match one point in the template (i.e., lengthening) and multiple points in the template can match one point in the sequence (i.e., shortening).

Dynamic time warping barycenter averaging inverts standard dynamic time warping: it discovers a latent invariant template from a set of sequences rather than identifying new instances of a known template. We used DBA to discover the short sequence of values that characterized the surprisal curves common to sentences of varying lengths. We first averaged individual sentences of the same length together and then applied the DBA algorithm to this set of average sequences.

We used the implementation of DBA in the Python package tslearn [23], which fit the barycenter to a time-series dataset through the expectation–maximization algorithm (EM) [24]. DBA in this implementation allowed us to specify the size of the barycenter. Because of the characteristic shape observed by Yu et al. [17] and found in our data (Figure 1), we chose a barycenter of length 5 to capture the varying information slopes across sentences. However, all of the results we report in this Study and in others were similar for barycenters of varying lengths.

### 2.2. Results and Discussion

We began by replicating Yu et al.’s analyses with a standard unigram model, examining the surprisal of words in sentence of length 15, 30, and 45 as they did. In line with their findings, we found a reliably non-linear shape in sentences of all 3 lengths, with the information in each word rising for the first two words, plateauing in the middle of sentences, dipping in the penultimate position, and rising steeply on the final word (Figure 2A). Qualitatively, we found the same shape in utterances of all other lengths we sampled, from utterances with 5 words to utterances with 45 words.

In comparison, under the trigram model, we observed 3 major changes. First, each word contained significantly less information. This was to be expected as knowing the two prior words made it much easier to predict the next word. Second, the fall and peak at the ends of the utterances was still observable, but much less pronounced. Finally, the first word of each sentence was now much more surprising than the rest of the words in the sentence, because the model had only the start-of-sentence token $\langle s\rangle$ to use as context. Thus, the trigram model likely overestimates the information for humans reading the first word. Together, these results suggest that Yu et al. [17] overestimated the non-uniformity of information in sentences. Nonetheless, the final words of utterances do consistently contain more information than the others.

Figure 2B shows the barycenters produced by the dynamic time warping barycenter averaging algorithm (DBA). It correctly recovers both the initial and final rise in information under the unigram model and the initial fall and smaller final rise in the trigram model. We took this as evidence that (1) these shapes were characteristic of all lengths, and (2) that DBA effectively recovered the characteristic information structure.

In sum, the results of Study 1 suggested that sentences of written English have a characteristic non-uniform information structure, with information rising at the ends of sentences. This structure is more pronounced when each word is considered in isolation, but some of the structure remains even when each word is considered in context. These results are broadly consistent with the predictions of a Uniform Information Density account: information increases over the course of sentences but approaches uniformity as more context is considered.

Is this structure unique to written English, or does it characterize spoken English as well? In Study 2, we applied this same analysis to two corpora of spoken English–the first of adults speaking to other adults, and the second of adults and children speaking to each other.

## 3. Study 2: Information in Spoken English

Spoken language is different from written language in several respects. First, the speed at which it can be processed is constrained by the speed at which it is produced. Second, speech occurs in a multimodal environment, providing listeners with information from a variety of sources beyond the words conveyed (e.g., prosody, gesture, world context). Finally, both words and sentence structures tend to be simpler as they must be produced and processed in real time [25]. Thus, sentences of spoken English may have different information curves than those of written English.

The language young children hear is further different from the language adults speak to each other. Child-directed speech tends to simpler in a number of dimensions including the lengths and prosodic contours of utterances, the diversity of words, and the complexity of syntactic structures [26]. The speech produced by young children is even more distinct from adult–adult speech, replete with simplifications and modifications imposed by their developing knowledge of both the lexicon and grammar [27]. In Study 2, we asked whether spoken English–produced both by adults and children–had the same characteristic information shape as written English.

### 3.1. Method

#### 3.1.1. Data

To estimate the information in utterances of adult–adult spoken English, we used the Santa Barbara Corpus of Spoken American English, a ∼250,000 word corpus of recordings of naturally occurring spoken interactions from different regions of the United States [28]. For parent–child interactions, we used all of the North American English corpora in the Child Language Data Exchange System (CHILDES) hosted through the childes-db interface [29,30]. We selected for analysis ∼1 million utterances produced by children (mostly under the age of five), and ∼1.7 million utterances produced by the parents of these children.

#### 3.1.2. Models

**Figure 3:**
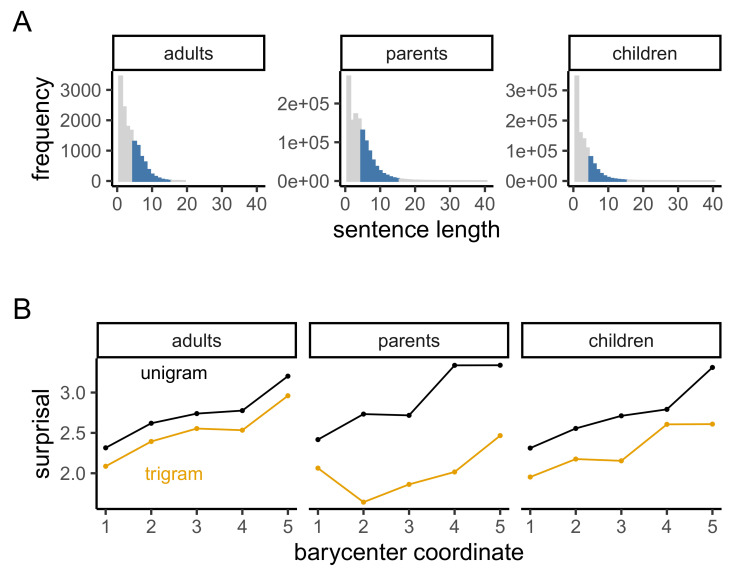
(**A**) The distribution of sentence lengths in the spoken English corpora: Adults in Santa Barbara, and parents and children in CHILDES. We analyzed sentences of length 5–15 (colored). (**B**) Characteristic surprisal curves for these corpora.

All pre-processing and modeling details were identical to Study 1 except for the selection of sentences for analysis. Because the utterances in both the Santa Barbara Corpus and CHILDES were significantly shorter than the sentences in the British National Corpus, we analyzed all utterances of at least 5 and most 15 words (see Figure 3A). Models were estimated separately for each of the 3 corpora.

### 3.2. Results and Discussion

The information curves found in adult–adult utterances were quite similar to those for parent–child and child–parent (Figure 3B). Under the unigram model, information rose steeply at the beginning of utterances, was relatively flat in the middle, and the rose even more steeply at the end. Under the trigram model, the curve was nearly identical. Both curves were similar in shape to the characteristic curve for written English estimated in Study 1.

Curves for parent and child speech were similar to those of adults with some small differences. Unigram curves for both monotonically increased although the largest jump in the parent curve occurred in the 4th barycenter position rather than the third. Trigram curves were also broadly similar although the parent curve dipped between the first and second position. This decreasing slope was inconsistent with the UID prediction of monotonic increase. It is possible that this decrease was a real feature of speech to children–parents may begin utterances to children more variably than when they speak to other adults.

Overall, the preponderance of evidence suggested that the characteristic shape of the information curve for spoken English is similar to that of written English, which appears to be approximately true in speech to and by children. All of these curves are broadly consistent with predictions from a UID account, according to which information measured without context should increase, and that including some context will reduce the rate of increase.

In Study 3 we applied this technique to a diverse set of written languages of different families to ask whether these structures vary cross-linguistically.

## 4. Study 3: Language Structures and Large-Scale Data Analysis

In Study 3, we applied the same method as in Studies 1 and 2 to a diverse set of over 200 natural languages represented on Wikipedia. In our prior studies, we found that the distribution of information in English sentences is broadly consistent with predictions of a Uniform Information Density account: information roughly rises over sequential words in a sentence, and further information rises more slowly when more prior context is used to predict the next word. In Study 3 we asked whether this same pattern characterizes natural languages in general and whether variability in the characteristic information curves of languages is related to their typological features.

### 4.1. Method

#### 4.1.1. Data

To measure cross-linguistic variation in the structure of information across sentences, we constructed a corpus of Wikipedia articles using the Wikiextractor tool from the Natural Language Text Analytics (TANL) pipeline [31]. We retained all languages with at least 10,000 articles, resulting in data from a set of 234 languages from 29 families.

To understand how potential variations in information curve shape are related to the structure of these languages, we used the available typological feature information in the World Atlas of Language Structures (WALS) [32], which has data for 144 typological features in thousands of languages. These features describe aspects of morphology, syntax, phonology, etymology and semantics–in short the features describe the structures in each language.

There are several categories of WALS features. Phonology describes sounds, stress, intonation, and syllable structure in each language. Nominal categories describe the morphology and semantics of nouns, including features for cases, definiteness and plurals. Verbal categories describe analogous verb features, focusing on tense, mood and aspect. Nominal syntax features describe a heterogeneous collection of noun phenomena, focusing on possessives and adjectives. Word order features describe not only canonical ordering of the subject, object and verb but also the orderings of heads and modifiers, relative clauses and other orderings. Simple clause features describe the syntax and organization of single clauses, such as passive and negative constructions.

#### 4.1.2. Models

Models were estimated separately for each language using the same procedures as in Study 1. To accommodate the variety of lengths across the language corpora, we analyzed sentences of 5–45 words, the boundaries of which were assumed to be identified by spaces as in Studies 1 and 2. The Wikipedia entries for all languages were hand-checked to the best of our abilities to ensure that this assumption was appropriate, but it is a potential source of noise in the analyses.

For each pair of languages, we derived two pairwise similarity measures. To estimate information structure similarity, we first centered each language’s 5-point barycenter curve (since surprisal highly correlates with corpus size), and then computed the cosine similarity between the two centered curves. To compare typological similarity, we computed the proportion of features in the WALS on which the two languages shared the same feature value. Because WALS is a compiled collection from the fieldwork of many linguists rather than a complete analysis by a single group, its features vary in the number of values they take. Some, like Vowel Quality Inventories (2A), have multiple features but also a natural ordering (small inventory, medium inventory, large inventory). However, others, like Position of Tense–Aspect Affixes (69A) have multiple values with no obvious ordering (prefixes, suffixes, tone, combination with no primary, none). For this reason, we used an exact match as our distance function, but other more sensitive metrics could be used by other researchers.

Finally, because of the collaborative construction of WALS, many features are missing in many languages. For this reason, only features present in WALS for both languages in a pair were considered for estimating their proportion of shared features. We also attempted to address this sparsity problem by imputing missing feature data where possible using multiple imputation with multiple correspondence analysis (MIMCA) [33]. It begins with mean imputation, converts the categorical WALS features into a numerical contingency table with dummy coding then repeatedly performs principle component analysis (PCA) and reconstructs the contingency table. Results of analyses using imputed features were qualitatively comparable to those performed on raw features, but with weaker correlations as described below. Nonetheless, we reported both measures as they may be of use in future research.

### 4.2. Results and Discussion

**Figure 4:**
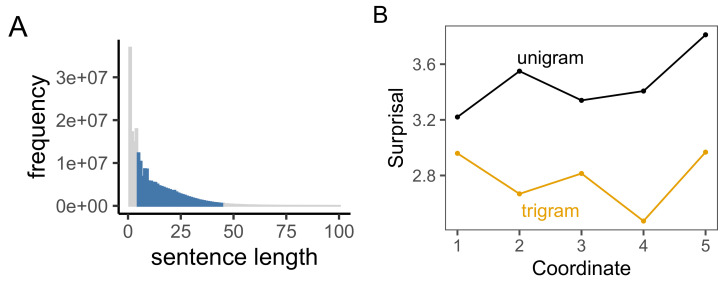
(**A**) The distribution of sentence lengths in the English Wikipedia Corpus. We analyzed sentences of length 5–45 (colored). (**B**) Characteristic information curves for English in the Wikipedia corpus.

In Studies 1 and 2, we observed that characteristic information curve of English to be generally increasing in both unigram and trigram models. In Study 3, we first replicated this analysis on the larger English language sample in the Wikipedia corpus (Figure 4). The unigram information curve for English estimated on Wikipedia was nearly identical to the curve observed for the British National Corpus in Study 1 (see Figure 2B). The trigram curve had a more pronounced dip in the 4th coordinate, but otherwise maintained the general shape and characteristic final rise we had previously observed.

This shape did not, however, characterize all of the 234 represented in Wikipedia. Languages varied widely in the shape of their information curves, both in the unigram and in the trigram model. Under the unigram model, some languages, like Spanish and German, were generally increasing like English. Others, like Hindi and Chinese, had negative slopes in their characteristic curves, while others like Urdu had a mix of positive and negative slopes. Under the trigram model, languages also varied, with reliable negative slopes in a number of languages including Russian and Urdu.

**Figure 5:**
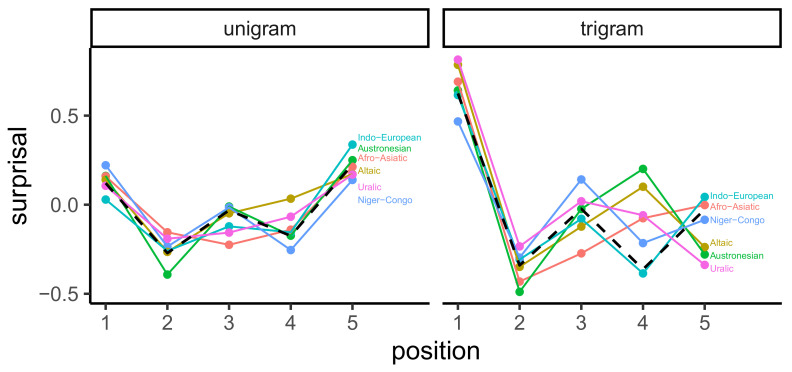
Centered characteristic information curves for the six most frequent language families represented on Wikipedia. The dashed line shows the mean curve across all language families.

In lieu of presenting characteristic curves for all languages, Figure 5 shows them for each of the six language families in which at least 10 languages were represented in Wikipedia. These curves were produced first by centering the surprisal for each language so that the surprisal of the mean word was 0, thereby deconfounding the differences due to corpus size, and then applying the barycenter averaging algorithm to the curves from all languages in the relevant family.

Two main trends were apparent. First, no language families had curves that were monotonically increasing, neither for the mean curve over all languages for either the unigram or the trigram model. Thus, the UID prediction did not appear to hold for languages other than English. Second, information curves for distinct families were more different from each other under the trigram model. To confirm this statistically, we computed the variance in surprisals at each of the 5 barycenter positions across all 234 languages. The average variance across the positions under the unigram model was 0.077 with a 95% confidence interval (0.05, 0.104). The average variance under the trigram model was 0.163 (0.113, 0.218). Matching our observation, these confidence intervals did not overlap.

**Figure 6:**
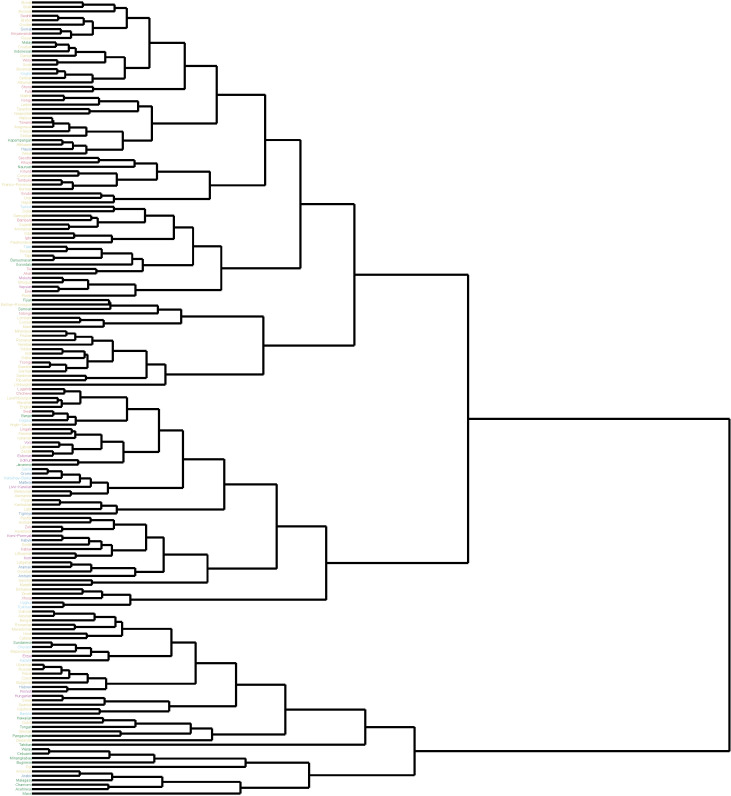
A dendrogram constructed by hierarchically clustering languages in the six most-well represented families on Wikipedia according to the similarities of their characteristic information curves. Languages are colored according to their language family.

Together, these results suggested that the characteristic information curves of different languages may diverge from the UID prediction because of the influence of idiosyncratic syntactic properties that prevent them from being optimal codes. If this is the case, then languages that are more similar phylogenetically may also have more similar information curves. Figure 6 shows a dendrogram produced by hierarchical clustering on the basis of the cosine similarity of information curves. All of the languages that belong from the families shown in Figure 5 are represented. Although not all members of each language family are clustered closely together, some structure is certainly apparent. For instance, all of the Austronesian languages are quite similar.

To test the hypothesis that linguistic similarity leads to information curve similarity, we considered the relationship between the typological features of languages and their characteristic information curves. For each pair of languages, we computed curve similarity using cosine distance, and typological similarity using the number of shared WALS features both raw and imputed. Under the unigram model, the two similarity measures were significantly but very weakly correlated ($r_{raw}\;=$ 0.056, $t_{raw}\;=$ 3.786, $p_{raw}$ < 0.001; $r_{imputed}\;=$ 0.025, $t_{imputed}\;=$ 2.715, $p_{imputed}\;=$ 0.007). Under the trigram model, this correlation was still low but an order of magnitude stronger ($r_{raw}\;=$ 0.203, $t_{raw}\;=$ 13.899, $p_{raw}$ < 0.001; $r_{imputed}=$ 0.124, $t_{imputed}\;=$ 13.349, $p_{imputed}$ < 0.001).

**Figure 7:**
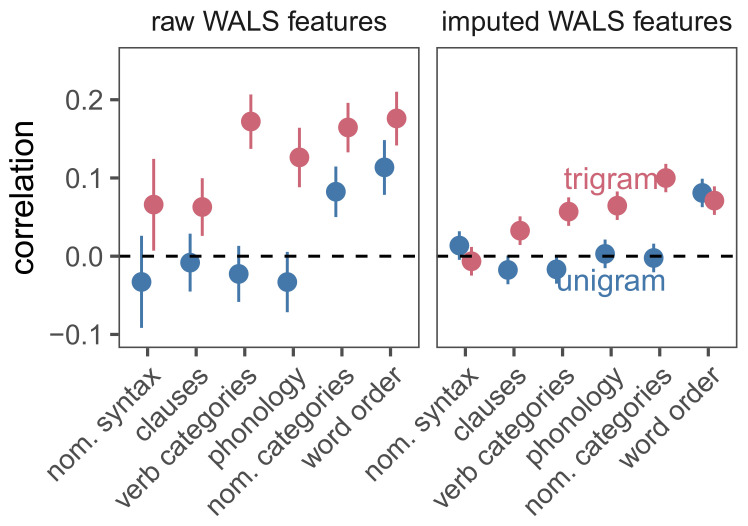
Pairwise correlations between languages’ normalized information curves and the number of linguistic features they share of each type. Error bars indicate 95% confidence intervals.

To understand which typological features contributed to these similarities, we split the WALS features by type such as nominative categories and nominative syntax describing morphology, while word order described subject–verb–object and head–modifier word orders. Figure 7 shows the correlation between the similarity of information curves under both the unigram and trigram models and the number of features of each of type that the two-languages shared. Under the unigram model, word order features, and possibly nominal category features, appeared to predict information curve similarity. In contrast, under the trigram model, all feature types except for possibly nominal syntax were reliably correlated with information curve similarity. Thus, almost all of the typological features represented in WALS were reflected in the characteristic information curves. These analyses suggest that languages may be pressured to be optimal codes, but that the historical influences on the structures of languages may put in place limits on the efficiency of these codes.

## 5. General Discussion

Why do languages have the regularities we observe? The particular features of any one language may owe their origin to a diverse set of ecological pressures, features of the population of speakers, or other idiosyncratic causes [34,35]. Despite the difficulty of explaining variability across languages, significant progress in explaining universals was made by taking an optimal coding perspective on language. If languages evolved to be efficient codes for transmitting information, they should all have certain predictable features [2,3].

One such predicted feature has been called Uniform Information Density–speakers should try to keep the amount of information they transmit constant rather than produce spikes that lead to difficulties in comprehension [7,8,9]. Genzel and Charniak [8] developed a clever method to confirm this prediction at the sentences level in an article, and a variety of other results have confirmed similar results at a variety of other language levels [11,36]. However, recent work from Yu et al. [17] suggested that this prediction may not hold at the level of subsequent words within a sentence.

In this paper, we built a method employed by Yu et al. [17] to develop a novel means of quantifying how information is typically distributed across the words of a sentence. We showed that English–whether written or spoken or produced by adults or children–has a prototypical information structure, which is broadly in line with the predictions of the Uniform Information Density Hypothesis.

However, the same prediction did not hold for many other languages. Instead, the characteristic information curves of languages are at least partially influenced by a variety of features of their typological structure (e.g., word order). These top-down constraints appear to place limits on the extent to which languages can approximate optimal codes. These results represent a small first step towards answering the question of how much these constraints shape a speaker’s production and how a speaker interacts with them.

## Data Availability

All data and code for this study are available in a public GitHub repository at https://github.com/jklafka/language-modeling (accessed on 26 September 2021).

## Abbreviations

The following abbreviations are used in this manuscript:

BNC	British National Corpus
SBC	Santa Barbara Corpus of Spoken American English
DBA	Dynamic Time Warping Barycenter Averaging
CHILDES	Child Language Data Exchange System
WALS	World Atlas of Linguistic Structure
MIMCA	Multiple Imputation Multiple Correspondence Analysis

## References

[1] Austin J.L. (1975). How to Do Things with Words.

[2] Shannon C.E. (1948). A mathematical theory of communication. Bell Syst. Tech. J..

[3] Anderson J.R., Milson R. (1989). Human memory: An adaptive perspective. Psychol. Rev..

[4] Kutas M., Federmeier K.D. (2011). Thirty years and counting: Finding meaning in the N400 component of the event-related brain potential (ERP). Annu. Rev. Psychol..

[5] Tanenhaus M.K., Spivey-Knowlton M.J., Eberhard K.M., Sedivy J.C. (1995). Integration of visual and linguistic information in spoken language comprehension. Science.

[6] Pickering M.J., Garrod S. (2013). An integrated theory of language production and comprehension. Behav. Brain Sci..

[7] Aylett M., Turk A. (2004). The smooth signal redundancy hypothesis: A functional explanation for relationships between redundancy, prosodic prominence, and duration in spontaneous speech. Lang. Speech.

[8] Genzel D., Charniak E. Entropy rate constancy in text. Proceedings of the 40th Annual Meeting of the Association for Computational Linguistics.

[9] Levy R., Jaeger T.F. (2007). Speakers optimize information density through syntactic reduction. Adv. Neural Inf. Process. Syst..

[10] Mahowald K., Fedorenko E., Piantadosi S.T., Gibson E. (2013). Info/information theory: Speakers choose shorter words in predictive contexts. Cognition.

[11] Jaeger T.F. (2010). Redundancy and reduction: Speakers manage syntactic information density. Cogn. Psychol..

[12] Frank A.F., Jaeger T.F. Speaking rationally: Uniform information density as an optimal strategy for language production. Proceedings of the Annual Meeting of the Cognitive Science Society.

[13] Piantadosi S.T., Tily H., Gibson E. (2011). Word lengths are optimized for efficient communication. Proc. Natl. Acad. Sci. USA.

[14] Kemp C., Regier T. (2012). Kinship categories across languages reflect general communicative principles. Science.

[15] Kirby S., Tamariz M., Cornish H., Smith K. (2015). Compression and communication in the cultural evolution of linguistic structure. Cognition.

[16] Perfors A., Navarro D.J. (2014). Language evolution can be shaped by the structure of the world. Cogn. Sci..

[17] Yu S., Cong J., Liang J., Liu H. (2016). The distribution of information content in English sentences. arXiv.

[18] Leech G.N. (1992). 100 Million Words of English: The British National Corpus (BNC).

[19] Chen S.F., Goodman J. (1999). An empirical study of smoothing techniques for language modeling. Comput. Speech Lang..

[20] Smith N.J., Levy R. (2013). The effect of word predictability on reading time is logarithmic. Cognition.

[21] Heafield K., Pouzyrevsky I., Clark J.H., Koehn P. Scalable modified Kneser-Ney language model estimation. Proceedings of the 51st Annual Meeting of the Association for Computational Linguistics.

[22] Petitjean F., Ketterlin A., Gançarski P. (2011). A global averaging method for dynamic time warping, with applications to clustering. Pattern Recognit..

[23] Tavenard R., Faouzi J., Vandewiele G., Divo F., Androz G., Holtz C., Payne M., Yurchak R., Rußwurm M., Kolar K. (2017). Tslearn: A Machine Learning Toolkit Dedicated to Time-Series Data. https://github.com/rtavenar/tslearn.

[24] Moon T.K. (1996). The expectation-maximization algorithm. IEEE Signal Process. Mag..

[25] Christiansen M.H., Chater N. (2016). The now-or-never bottleneck: A fundamental constraint on language. Behav. Brain Sci..

[26] Snow C.E. (1972). Mothers’ speech to children learning language. Child Development.

[27] Clark E.V. (2009). First Language Acquisition.

[28] Du Bois J.W., Chafe W.L., Meyer C., Thompson S.A., Martey N. (2000). Santa Barbara Corpus of Spoken American English.

[29] MacWhinney B. (2000). The CHILDES Project: The Database.

[30] Sanchez A., Meylan S.C., Braginsky M., MacDonald K.E., Yurovsky D., Frank M.C. (2019). Childes-db: A flexible and reproducible interface to the Child Language Data Exchange System. Behav. Res. Methods.

[31] Attardi G., Dei Rossi S., Simi M. The tanl pipeline. Proceedings of the Workshop on Web Services and Processing Pipelines in HLT.

[32] Dryer M.S., Haspelmath M. (2013). WALS Online.

[33] Audigier V., Husson F., Josse J. (2017). MIMCA: Multiple imputation for categorical variables with multiple correspondence analysis. Stat. Comput..

[34] Maddieson I., Coupé C. (2015). Human spoken language diversity and the acoustic adaptation hypothesis. J. Acoust. Soc. Am..

[35] Lupyan G., Dale R. (2010). Language structure is partly determined by social structure. PLoS ONE.

[36] Van Son R.J., van Santen J.P. (2005). Duration and spectral balance of intervocalic consonants: A case for efficient communication. Speech Commun..

